# Pulmonary Tuberculous: Symptoms, diagnosis and treatment. 19-year experience in a third level pediatric hospital

**DOI:** 10.1186/1471-2334-14-401

**Published:** 2014-07-19

**Authors:** Napoleón González Saldaña, Mercedes Macías Parra, Marte Hernández Porras, Pedro Gutiérrez Castrellón, Valeria Gómez Toscano, Hugo Juárez Olguin

**Affiliations:** 1 Servicio de Infectología, Instituto Nacional de Pediatría, (INP), Mexico City, Mexico; 2 Departamento de Investigación, INP, Mexico City, Mexico; 3 Laboratorio de Farmacología, INP, Facultad de Medicina, Universidad Nacional Autónoma de México, Av Imán #1, 3er piso, Col Cuicuilco, CP 04530 Mexico City, Mexico

**Keywords:** Tuberculosis, Pulmonary tuberculosis, Tuberculosis in children

## Abstract

**Background:**

Pulmonary tuberculosis (PTB) is an infectious disease that involves the lungs and can be lethal in many cases. Tuberculosis (TB) in children represents 5 to 20% of the total TB cases. However, there are few updated information on pediatric TB, reason why the objective of the present study is to know the real situation of PTB in the population of children in terms of its diagnosis and treatment in a third level pediatric hospital.

**Methods:**

A retrospective study based on a revision of clinical files of patients less than 18 years old diagnosed with PTB from January 1994 to January 2013 at Instituto Nacional de Pediatria, Mexico City was carried out. A probable diagnosis was based on 3 or more of the following: two or more weeks of cough, fever, tuberculin purified protein derivative (PPD) +, previous TB exposure, suggestive chest X-ray, and favorable response to treatment. Definitive diagnosis was based on positive acid-fast bacilli (AFB) or culture.

**Results:**

In the 19-year period of revision, 87 children were diagnosed with PTB; 57 (65.5%) had bacteriologic confirmation with ZN staining or culture positive (in fact, 22 were ZN and culture positive), and 30 (34.5%) had a probable diagnosis; 14(16.1%) were diagnosed with concomitant disease, while 69/81 were immunized. Median evolution time was 21 days (5–150). Fever was found in 94.3%, cough in 77%, and weight loss in 55.2%. History of contact with TB was established in 41.9%. Chest X-ray showed consolidation in 48.3% and mediastinal lymph node in 47.1%. PPD was positive in 59.2%, while positive AFB was found in 51.7% cases. Culture was positive in 24/79 patients (30.4%), PCR in 20/27 (74.1%). 39 (44.8%) patients were treated with rifampin, isoniazid, and pyrazinamide while 6 (6.9%) received the former drugs plus streptomycin and 42 (48.3%) the former plus ethambutol. There were three deaths.

**Conclusions:**

PTB in pediatric population represents a diagnostic challenge for the fact that clinical manifestations are unspecific and the diagnosis is not confirmed in all cases; that is why clinical suspicion, X-ray findings and PPD are indispensable for opportune start of treatment.

## Background

Pulmonary tuberculosis (PTB) is an infectious disease caused by *Mycobacterium tuberculosis.* This microorganism not only infects the lung but also other organs such as brain, kidneys and lymph nodes.

Today, tuberculosis constitutes global public health problem with a greater impact in less industrialized countries. According to the estimates of World Health Organization (WHO), there are around 8.7 million new cases every year out of which 0.5 million are children [1]. The Pan American Health Organization (PAHO) notifies 250,000 cases each year with a yearly toll of around 20,000 deaths [2]. It is estimated that in countries of low endemicity, tuberculosis in pediatric age represents less than 5% of all cases of tuberculosis while in high endemic countries; it could be as high as 20% [3].

Morbidity and mortality associated with TB are greater in developing nations where 95% of all cases and 98% of all deaths associated with TB were reported to occur in 1990 [4]. In Mexico, an increase of 28% in the number of cases of tuberculosis was reported from 1984 to 2001, with 14,612 in 1984 and 18,746 in 2001 notified to the General Direction of Epidemiology. However, in the subsequent years (2002–2005) this tendency was reverted pointing to a decrease in the number of cases with a report of 16,336 cases in 2005. Nevertheless, this decline in the cases did not last long for the fact that from 2006 to 2011, there was an increase, although gradual, in the number of cases that culminated to a report of 18,984 cases in 2011. 81.4% of this number was pulmonary tuberculosis and 3.1% of all the cases were notified in children under 15 years old [5,6].

Tuberculosis in pediatric age usually indicates an unidentified and untreated tuberculous contact. The hindrance in establishing the diagnosis is mainly due to its nonspecific symptoms and lack of possible microbiological confirmation. These could explain why the cases of this infection are under-reported in pediatric age. For this, it is fundamental to highlight the importance of active search through epidemiological and clinical histories, and microbiological studies when there are nonespecific symptoms such as fever and cough in this age group [7]. Even though some patients have an underlying disease, severe malnutrition should not be forgotten as a predisposing factor to acquire the disease [8]. It has been reported that the presence of infection by human immunodeficiency virus (HIV) is a factor that increases the cases of tuberculosis. In Mexico, the association of HIV to TB is only 5.8% in pediatric population and 8% in adults. In spite of these figures the country is still considered as a moderately TB endemic place. In other developing countries such as Peru and Brazil, an incidence of TB/HIV association of 1.5% and 42% was reported respectively [9].

The above led to the derivation of the objective of this study to evaluate the clinical, radiological, microbiological and immunological spectra of pulmonary tuberculosis seen in children hospitalized in a third level pediatric hospital of Mexico City.

## Methods

A retrospective and descriptive study based on a review of the clinical files of children less than 18 years old hospitalized from 1994 to 2013 at National Institute of Pediatrics (NIP) with diagnosis of pulmonary tuberculosis on discharge were carried out. Patient information on the ailment were obtained based on diagnosis of TB and PTB on admission and on discharge as well as on a review of all samples sent to the Microbiology Department in this period for ZN or culture for *M. tuberculosis*. This study was approved by the ethics committee of National Institute of Pediatrics (NIP).

NIP is a reference hospital with a yearly average of about 6,000 admissions of children less than 18 years old From Mexico city and other states of the republic. A probable case of PTB is considered when there are 2 or more of the following symptoms: 2 or more weeks of cough; chest X-ray suggestive of PTB (2 or more weeks of consolidation without any response to antibiotics, mediastinal lymphadenopathies, miliary pattern, calcifications, pleural effusion or caverns based on official report by a radiologist of NIP radiology department); positive PPD (≥5 mm in patients under 5 years old and immunocompromised, and of ≥10 mm for patients above this age) after 48 to 72 h of intradermal administration of 5 units/0.1 ml of tuberculin RT23 [3]; positive epidemiological contact of tuberculosis; histopathological findings consistent with tuberculosis; or favorable response to antituberculous treatment. The later was based on isoniazid, rifampin, and pyrazinamide from 1994 to 1997, but due to questions of resistance to rifampin, a new scheme of four drugs comprising of the addition of streptomycin or ethambutol to the former emerged. Also, under this consideration are patients with clinical and/or radiological diagnosis. The results of polymerase chain reaction (PCR) for tuberculosis were registered. Definitive diagnosis includes those patients with positive ZN staining or culture Lowenstein Janssen agar and Middlebrook 7H9. The history of BCG application confirmed with records of immunization was analyzed. From the statistical point of view, data were analyzed with the statistical program SPSS version13.0. Categorical variables by percentages and continuous variables with a median and a minimum-maximum value were described. Chi square test or Fisher´s exact test was used to compare Categorical variables, while T student or U Mann Whitney test was used for comparison of continuous variables of two means and One-way ANOVA or Kruskal Wallis test in the case of more than two means. Differences were considered statistically significant when p ≤ 0.05.

## Results

**Figure 1:**
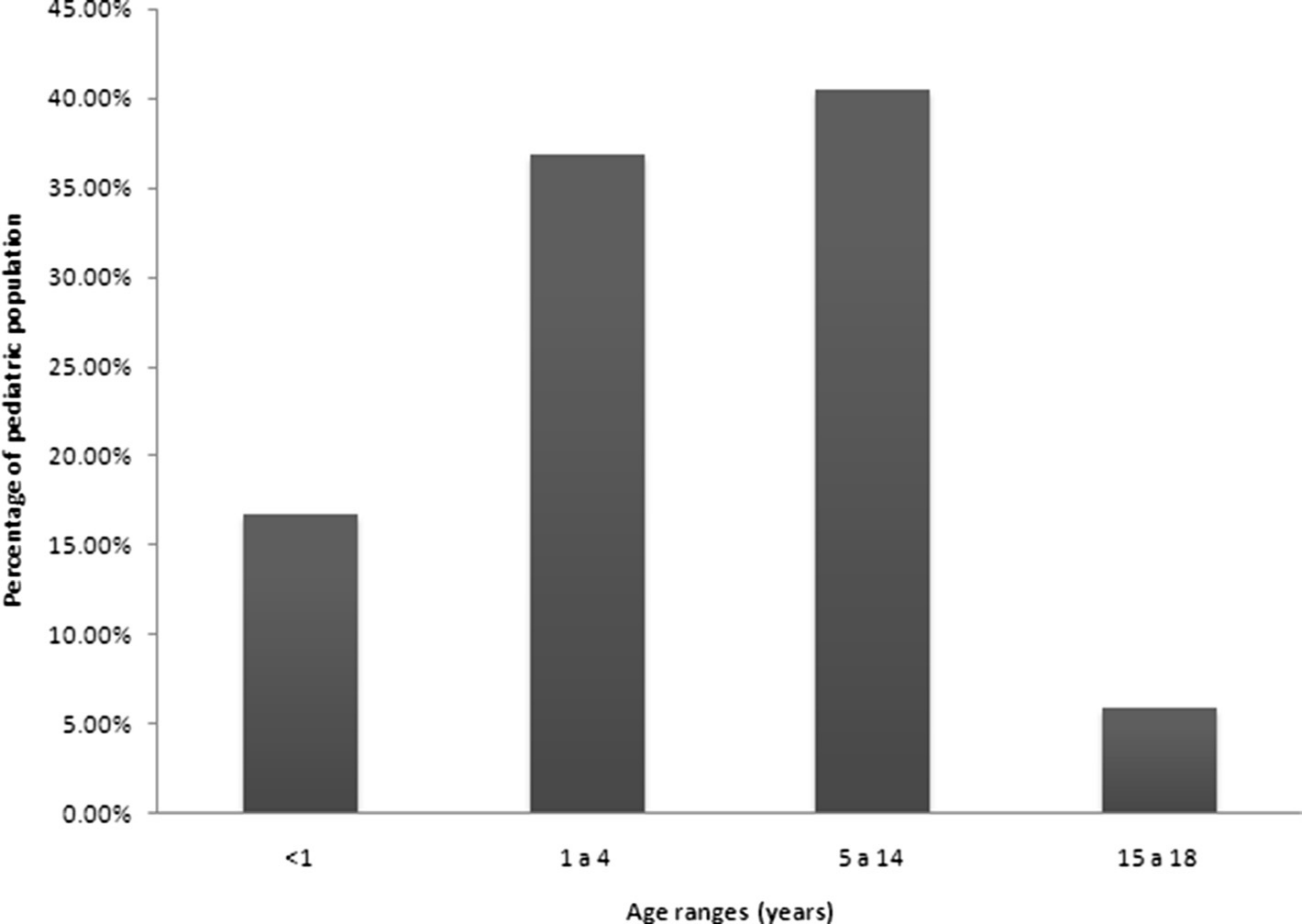
Distribution by age of children with pulmonary tuberculosis.

On application of the inclusion criteria, a probable case of tuberculosis were found in a total of 1,601subjects in the period studied. Nevertheless, only 87 (5.4%) of this total were diagnosed with PTB. The rest of the cases were diagnosed to have either extra pulmonary, systemic, or miliary tuberculosis. 57 (65.5%) of the PTB cases were classified as definitive and 30 (34.5%) as probable. From 1994 to 2000, an annual average of 9.4 cases was registered, while from 2001 to 2005, this average was 3.3 cases. 2006 to 2013 witnessed a decrease in the annual average with 1.4 cases. Of the total cases of PTB, 48 patients (55.2%) were male, while their median age was 3 years (3 months to 17 years). Eight patients (9.2%) had mild acute malnutrition, 15 (17.2%) had severe malnutrition, and the rest 64 patients (73.5%) were eutrophic. More than 70% of the study group was from 1 to 14 years old (Figure 1). There was an underlying disease in 14 (16.1%) of the PTB patients distributed as follows: systemic lupus erythematous 3, osteosarcoma 3, HIV 2, lymphoma 2, chronic granulomatous disease 2, ataxia telangiectasia 1, Wilms tumor 1; and 17.2% had severe malnutrition according to Waterlow classification. In terms of origin, 47 (54%) of the patients were from Mexico City and State of Mexico, while the rest 40 (46%) were from other parts of the republic. BCG application history was found in 81 patients with PTB while immunization was documented in 69 (85.2%). Epidemiological contact with tuberculosis was recorded in 86 (98.9%) of PTB cases with identification of 36 (41.9%) positive cases. Of this number of positive cases, 75% of the contact came from the “care-taker”. The median time of evolution was 21 days (5 to 150 days), and the predominant symptoms were fever, cough and weight loss. PPD was applied to 71 patients with 42 (59.2%) positive results. The most frequent radiological findings were either lobar or segmental consolidation (48.3%) and mediastinal lymph nodes (47.1%). These mediastinal lymph nodes were localized as follows: hilar 70%, peritracheal 27% and intercarinae 3%. Median (min-max) hemoglobin (g/dL), leucocytes (mm^3^), and platelets (mm^3^) in the whole sample were 10.8 (6–15); 11,100 (1,700-33,100), and 320,000 (13,700-1,240,000), respectively. Bacteriological confirmation was done in 83 patients (95.4%), out of whom 58 (66.7%) were in gastric lavage, 25(28.7%) in sputum, and 4 (4.6%) in bronchial lavage; culture for *M. tuberculosis* was done in 79 patients (90.8%) with a positive outcome in 24 cases (30.4%). PCR for *M. tuberculosis* was documented in 27 patients with 12 of them in sputum, 10 in gastric lavage, and 5 in bronchial lavage. Of the total PCR, 20 (74.1%) were positive. In 6 patients with positive culture for mycobacterium in whom PCR was also done, 5 (83.3%) were positive.

The antituberculous treatment applied to 39 (44.8%) of the patients from 1994 to 1997 were isoniazid, rifampin, and pyrazinamide while from 1998 to 2005 and due to questions of resistance to rifampin, a new scheme of four drugs comprising of the addition of streptomycin or ethambutol to the former came into use as was commented previously. This new scheme was applied to 48 (55.2%) of PTB cases with 6 of them receiving streptomycin while the rest (42) received ethambutol. From the patients who received ethambutol, 26 were under 6 years old. The duration of treatment was 6 months in 46 patients, 9 months in 19 patients, 12 months in 16 children, 18 months in 3 cases, and 24 months in only 1 case. Treatment was well tolerated in most of the cases. There were only 3 deaths (3.5%) one of which is a patient with HIV who crossed with multiple drug resistant PTB and poor treatment compliance. The other 2 patients died for causes not related to PTB. The rest of the patients had remission of their symptoms with improvement or radiological remission at the end of the treatment. In no case was there a request of AFB or culture controls and monitoring was just for 3 to 6 months after treatment. Therefore, it is unknown whether there was or no relapse or reinfection.

On comparison of epidemiological, clinical, radiological, and bacteriological variables between patients with definitive versus probable PTB, we identify meaningful differences in the size of PPD, 26 (72.2%) vs. 10 (27.8%) cases, with statistical difference, and the number of children with consolidation in chest X-ray 33 (78.6%) vs. 9 (21.4%), statistical difference (p < 0.05). This difference in relation to the history of BCG was meaningful between the two groups 41 (59.4%) vs. 28 (40.6%) for definitive cases and probable cases of PTB respectively (Table 1) with statistical difference. The 4 patients had lobar consolidation with one of them having pleural discharge and another with cavern.

**Table 1:** Comparison between cases of definitive VS probable pulmonary tuberculosis

**Variable**	**Definitive PTB**		**Probable PTB**	
	***N***	***(%)***	***n***	***(%)***
History of BCG +*	41	(59.4%)	28	(40.6%)
Combe +*	26	(72.2%)	10	(27.8%)
PPD +	9.5	(0–30)	3.0	(0–15)
Expectoration	16	(80%)	4	(20%)
Hemoptysis	4	(80%)	1	(20%)
Consolidation*	33	(78.6%)	9	(21.4%)
Mediastinal lymph nodes	26	(63.4%)	15	(36.6%)
Calcifications	10	(66.7%)	5	(33.3%)
Pleural effusion	10	(71.4%)	4	(28.6%)
Caverns	6	(85.7%)	1	(14.3%)
Miliary image	3	(60%)	2	(40%)

*Statistical difference p < 0.05.

Finally, a comparative analysis between the variables of interest in relation to the different age groups defined in Figure 1 at the beginning of the analysis was done; identifying statistically significant differences in relation to expectoration, hemoptysis, consolidation, caverns, calcification, mediastinal lymph nodes, AFB, and with pleural effusion (Table 2).

**Table 2:** Symptomatology and radiological finding in 87 patients with pulmonary tuberculosis

**Variable**	**<1 year (y)** ***n (%)***	**1-4 y** ***n (%)***	**5-14 y** ***n (%)***	**15-17 y** ***n (%)***	**Total**
History of BCG +	12 (92.3%)	26 (83.9%)	27 (79.4%)	4 (80%)	69 (79.3%)
Previous TB exposure	8 (61.5%)	11 (35.5%)	14 (41.2%)	3 (60%)	36(41.3%)
PPD +	6(45.5)	20(64)	26(78.3)	2(40)	54(62%)
Fever	14 (100%)	30 (96.8%)	33 (97.1%)	5 (100%)	82 (94.3%)
Cough	12 (85.7%)	23 (74.2%)	27 (79.4%)	5 (100%)	67 (77.0%)
Weight loss	8 (57.1%)	19 (61.3%)	17 (50%)	4 (80%)	48 (55.2%)
Expectoration*	1 (7.1%)	5 (16.1%)	9 (26.5%)	5 (100%)	20 (22.9%)
Hemoptysis*	0 (0%)	0 (0%)	4 (11.8%)	1 (20%)	5 (5.7%)
Consolidation*	2 (14.3%)	16 (51.6%)	21 (61.8%)	3 (60%)	42 (48.3%)
Mediastinal lymph nodes*	12 (85.7%)	15 (48.4%)	13 (38.2%)	1 (20%)	41 (47.1%)
Calcifications*	1 (7.1%)	4 (12.9%)	8 (23.5%)	2 (40%)	15 (17.2%)
Pleural effusion*	0 (0%)	8 (25.8%)	5 (14.7%)	1 (20%)	14 (16.1%)
Caverns*	0 (0%)	0 (0%)	5 (14.7%)	2 (40%)	7 (8.1%)
Miliary image	2 (14.3%)	2 (6.5%)	1 (2.9%)	0 (0%)	5 (5.7%)
AFB + in general*	4 (30.8%)	15 (48.4%)	21 (61.8%)	5 (100%)	45(51.7%)
AFB + in sputum			14 (41.2%)	4 (80%)	18(20.6%)
AFB + in gastric lavage	4 (30.8%)	15 (48.4%)	6 (17.6%)	2 (40%)	27(31%)
Culture +	3 (23.1%)	7 (22.6%)	12 (35.3%)	2 (40%)	24(27.5%)

*Statistical difference (p < 0.05).

## Discussion

In this study, PTB frequency was found to be 5.4% of all the forms of tuberculosis. This markedly contrasts with 81.4% found in the general population in our country and with 80% of cases published in other pediatric series [10,11]. This could be explained based on inclusion of only children without clinical signs of extra pulmonary disease and for the fact that PTB patients are only hospitalized when they present the severe form of the disease. It is worthwhile to point out that in this age group, the systemic form of the disease is important, reason why the exclusion drastically affected the frequency.

In this study, a greater percentage of PTB case (51.7%) was found in less than five-year old group which drastically reduced in older patients above 15 years. This coincides with 60% reported in the literature for less than 15-year-old patients indicating that in pediatric population, the risk of developing the disease is greater in the first years of life [12]. We did not find significant differences in age-disease distribution among the patients however; the period covering the first 6 years witnessed a greater number of cases with a reduction of up to 85% in the following years. This could explain the declining number of cases reported in the general population.

Even though acquired immunodeficiencies constitute a risk factor for the development of tuberculosis, in our series, only two cases had HIV (2.3%), which is comparable to nearly 3% reported in developed countries [9,13] and sharply contrasts with 12 to 37% reported in developing countries [14,15]. The explanation of this is farfetched, even when it has been reported that in adult patient with HIV, reactivation constitutes the most important pathogenic mechanism of tuberculosis, and that in countries with less prevalence of the disease, tuberculosis in general is acquired at later ages and reactivation is a very rare event in children, which could explain the low prevalence of HIV in this series [10].

Epidemiological contact of TB was found in 41.9% of our PTB cases with children less than one-year old accounting for 52.8% of the cases but with lesser contacts established. As usual, the source of infection was associated in most cases (75%) to the “caretaker”. The percentage of familiar contacts was significantly less in children of 15-year old and more due to increased extra domiciliary contacts in this age group. However, in the present series, we did not find such contacts, similar to what is reported in other series [16].

The protective clinical efficacy of BCG vaccine has been a matter of controversy, although it is accepted that this is greater for miliary and meningeal TB than for pulmonary form [17,18]. In this study, it is important to note the tendency of greater number of positive cultures in patients with history of BCG immunization, where we supposed to have a lesser mycobacterial replication and in consequence a lesser number of bacilli.

In our series, all the patients were symptomatic when the diagnosis was made, similar to that described by Vallejo et al. in Texas, where in a series of 47 infants, 79% of the cases, were diagnosed by the presence of symptoms, and only19% was by epidemiological history [16]. However, this data contrasts with the reports of Sánchez-Albisua et al. in Madrid, where in a series of 173 children under 15 years, 59% of the cases were identified by contact [17], probably in relation to an active search of the contacts, situation that allows the identification of cases in very early stages of the disease [19].

We found nonspecific signs and symptoms with fever and cough being the most consistent clinical manifestations, independently of age, which account for 94.3% of the reason for medical consult. Nonetheless, loss of weight was seen in most of the patients independently of the age. Expectoration and hemoptysis were significantly more frequent in the group older than 14 years, which is similar to the findings in adults with PTB [20]. PPD was positive in an important proportion of the cases (59.2%), mostly in children older than 1 year. Again, this is similar to that reported by other authors (35-60%), probably due to inclusion of only patients with PTB in our series which is different from other series where different forms of tuberculosis (extra pulmonary or disseminated) were included with positive PPD of 32% to 50% [21,22].

It is of interest to note that in this study PPD was negative in 2 (including a patient with positive culture) of the 3 patients older than 15 years with normal state of nutrition and one with an identified anergizing disease which could be attributed to lack of PPD response as stated by other authors who found lack of response to PPD in approximately 10% of the patients without any underlying disease with tuberculosis documented by culture [21].

Radiological studies play an important role in the diagnosis of tuberculosis in pediatric age. Although there can be some differences in its interpretation, the findings suggestive of PTB in children under 5 years old are of great support for its diagnosis. In this series, the image of consolidation was a frequent finding in all age groups and was significantly more frequent in patients older than 5 years, in contrast with mediastinal lymphadenopathies, which were present in children below this age coinciding with what was reported by other authors. The miliary pattern was seen in only 6.9% of the cases, mainly in patients below 5 years old, with a clear prevalence in those less than 1 year old, in whom the disseminated disease is more frequent. Pleural effusion is infrequent in pediatric age but in our series it was seen in 14.3% of the cases [23]. The presence of calcifications and caverns were significantly more frequent in patients above 14 years old due to the fact that it is an infrequent complication of primary tuberculosis [10,12,16,21-23]. It has been identified that the presence of fever and cough of 2 or more weeks, plus a positive PPD of 10 or more millimeters, have a positive predictive value of 73% with a sensitivity of 44% for tuberculosis confirmed by culture [24]. Based on this, it was suggested that children fulfilling 2 of these 3 criteria should be evaluated with chest X-ray and ZN positive [24,25].

The microbiological diagnosis is denoted with difficulties because the sensitivity of ZN positive is from 20 to 25%. In this study however, ZN was positive in 51.7%. With respect to this, it is important to note that this was performed in children older than 5 years old in whom PTB forms such as caverns contain a greater number of mycobacteria in contrast with the type of lesions found in children less than this age where the TB is habitually paucibacillary. Mycobacterium identified by culture is present in 30.4% of the cases. These results are comparable to variations in different reports, where culture was found positive in 20 to 75% of cases [20,26,27]. PCR value in diagnosis of tuberculosis in pediatrics is still controversial due to the false positives, mainly in countries with high endemicity for tuberculosis, and to the false negatives that are reported. In this study it was positive in 83.3% of the cases documented by culture [28-30].

Antituberculous treatment was well tolerated in most cases. Patients responded well to the treatment even when there was one death, precisely a boy with HIV and multidrug resistant tuberculosis without adherence to treatment. The other 2 deaths were not attributable to tuberculosis. 54.1% of the children were treated for 6 months with clinical and radiological response similar to those who were treated from 9 to 24 months (treatment for such long periods of time was administered in patients with underlying immunosuppressive diseases), without clinical evidence of related complications.

## Conclusion

Diagnosis of PTB in pediatric population represents a challenge due to nonspecific nature of its signs and symptoms and to the low possibility of identification of *Mycobacterium tuberculosis*, as we found in this series of cases (only in 30.4%). So, clinical suspicion, chest X-ray findings, and contact study with the aim of establishing a timely treatment are fundamental in its diagnosis. It is important to say that monitoring the clinical evolution is fundamental in all cases, given that in an important number of them, treatment is indicated without microbiological correlation.

## Competing interests

The authors declare that they have no competing interests.

## Authors’ contributions

NGS, MMP, and MHP have made substantial contributions to conception and design, acquisition of data and analysis, and interpretation of the same. PGC, VG and HJO were involved in drafting the manuscript, and its critical review for important intellectual content. All authors read and approved the final manuscript.

## Pre-publication history

The pre-publication history for this paper can be accessed here:

http://www.biomedcentral.com/1471-2334/14/401/prepub

## References

[B1] World Health Organization Global tuberculosis report 2012 http://www.who.int/tb/publications/global_report/en/

[B2] Pan American Health Organization Epidemiological evaluation of tuberculosis. Tendencies in some countries of the Americas Bol Epidemiol 1987 8 1 5

[B3] Walls T Shingadia D Global epidemiology of paediatric tuberculosis J Infect 2004 48 13 22 10.1016/S0163-4453(03)00121-X 14667788

[B4] Raviglione MC Snider DE Jr Kochi A Global epidemiology of tuberculosis: morbidity and mortality of a worldwide epidemic JAMA 1995 273 220 226 10.1001/jama.1995.03520270054031 7807661

[B5] General Direction of Epidemiology Unified information system for the epidemiological surveillance/SSA 2012 http://www.epidemiologia.salud.gob.mx/dgae/infoepid/vig_epid_manuales.html

[B6] Annuals of morbidity and mortality Unified information system for the epidemiological surveillance 1984-2011/SSA: http://www.epidemiologia.salud.gob.mx/dgae/infoepid/inicio_anuarios.html

[B7] Stop TB. Partnership Childhood TB Subgroup World Health Organization Guidance for national tuberculosis programmes on the management of tuberculosis in children. Chapter 1: Introduction and diagnosis of tuberculosis in children Int J Tuberc Lung Dis 2006 10 1091 1097 17044200

[B8] Waterlow JC Note on the assessment and classification of protein-energy malnutrition in children Lancet 1973 14;2 7820 87 89 10.1016/s0140-6736(73)93276-5 4123633

[B9] Pan-American Health Organization Tuberculosis in the Americas 2012 Regional report 2013 Washington, DC: Epidemiology, control y finance

[B10] Sánchez-Albisua I Baquero-Artigao F Del Castillo F Borque C García-Miguel MJ Vidal ML Twenty years of pulmonary tuberculosis in children: what has changed? Pediatr Infect Dis J 2002 21 49 53 10.1097/00006454-200201000-00011 11791099

[B11] Kimerling ME Vaughn ES Dunlap NE Childhood tuberculosis in Alabama: epidemiology of disease and indicators of program effectiveness, 1983 to 1993 Pediatr Infect Dis J 1995 14 678 684 10.1097/00006454-199508000-00006 8532425

[B12] Starke JR Taylor-Watts KT Tuberculosis in the pediatric population of Houston, Texas Pediatrics 1989 84 28 35 2500637

[B13] Chan SP Birnbaum J Rao M Steiner P Clinical manifestation and outcome of tuberculosis in children with acquired immunodeficiency syndrome Pediatr Infect Dis J 1996 15 443 447 10.1097/00006454-199605000-00012 8724068

[B14] De Cock KM Soro B Coulibaly IM Lucas SB Tuberculosis and HIV infection in sub-Saharan Africa JAMA 1992 268 1581 1587 10.1001/jama.1992.03490120095035 1518113

[B15] Chintu C Bhat G Luo C Raviglione M Diwan V Dupont HL Zumla A Seroprevalence of human immunodeficiency virus type 1 infection in Zambian children with tuberculosis Pediatr Infect Dis J 1993 12 499 504 10.1097/00006454-199306000-00008 7688450

[B16] Vallejo JG Ong LT Starke JR Clinical features, diagnosis and treatment of tuberculosis in infants Pediatrics 1994 94 1 7 8008511

[B17] Sánchez-Albisua I Vidal López ML del Castillo MF Borque C García-Miguel MJ García-Hortelano J Pulmonary tuberculosis in children: its age-dependent aspects An Esp Pediatr 1998 48 251 255 9608084

[B18] Colditz GA Brewer TF Berkey CS Wilson ME Burdick E Fineberg HV Mosteller F Efficacy of BCG vaccine in the prevention of tuberculosis. Meta-analysis of the published literature JAMA 1994 271 698 702 10.1001/jama.1994.03510330076038 8309034

[B19] Marais BJ Gie RP Hesselinc AC Schaaf S Lombard C Erarson DA Beyer N A defined symptom-based approach to diagnose pulmonary tuberculosis in children Pediatrics 2006 118 e1350 e1359 10.1542/peds.2006-0519 17079536

[B20] Seth V Singhal PK Semwal OP Kabra SK Jain Y Childhood tuberculosis in a referral centre: clinical profile and risk factors Indian Pediatr 1993 30 479 485 8288329

[B21] Starke JR Diagnosis of tuberculosis in children Pediatr Infect Dis J 2000 19 1095 1096 10.1097/00006454-200011000-00015 11099094

[B22] Dogru D Ozcelik U Gocmen A Pediatric primary pulmonary tuberculosis Chest 2002 121 1722; 1434 1438 12006474 10.1378/chest.121.5.1722

[B23] Schaaf HS Beyers N Gie RP Nel ED Smuts NA Scott FE Donald PR Fourie PB Respiratory tuberculosis in childhood: the diagnostic value of clinical features and special investigations Pediatr Infect Dis J 1995 14 189 194 10.1097/00006454-199503000-00004 7761183

[B24] Salazar GE Schmitz TL Cama R Sheen P Franchi LM Centeno G Varela C Leyva M Montenegro-James S Overhelman R Gilman RH Thompson MJ Working Group on TB in Peru Pulmonary tuberculosis in children in a developing country Pediatrics 2001 108 448 453 10.1542/peds.108.2.448 11483814

[B25] Castán Vidal ML Vidal López ML Cerro Marín MJ Rey Durán R Ortega Calderón A García-Hortelano J Contacts of children with tuberculous patients An Esp Pediatr 1991 34 129 131 2042805

[B26] Driver CR Luallen JJ Good WE Valway SE Frieden TR Onorato IM Tuberculosis in children younger than five years old: New York City Pediatr Infect Dis J 1995 14 112 117 10.1097/00006454-199502000-00006 7746692

[B27] Starke JR Childhood tuberculosis: A diagnostic dilemma Chest 1993 104 329 330 10.1378/chest.104.2.329 8339609

[B28] Smith KC Starke JR Eisenach K Ong LT Denby M Detection of *Mycobacterium tuberculosis* in clinical specimens from children using a polymerase chain reaction Pediatrics 1996 97 155 160 8584370

[B29] Delacourt C Poveda JD Chureau C Beydon N Mahut B de Blic J Scheinmann P Garrigue G Use of polymerase chain reaction for improved diagnosis of tuberculosis in children J Pediatr 1995 126 703 709 10.1016/S0022-3476(95)70396-9 7751992

[B30] Migliori GB Borghesi A Rossanigo P Adriko C Neri M Santini S Bartoloni A Paradisi F Acocella G Proposal of an improved score method for the diagnosis of pulmonary tuberculosis in childhood in developing countries Tuber Lung Dis 1992 73 145 149 10.1016/0962-8479(92)90148-D 1421347

